# Does negative voice content enhance strong priors and conditioned hallucinations?

**DOI:** 10.1192/j.eurpsy.2025.2051

**Published:** 2025-08-26

**Authors:** C. Jacquet, C. Dondé, C. Bortolon

**Affiliations:** 1 38000, Univ. Grenoble Alpes, Univ. Savoie Mont Blanc, LIP/PC2S; 2 38000, Centre Hospitalier Universitaire Grenoble Alpes, GIN, Grenoble; 3 38120, Centre Hospitalier Alpes Isère, Saint-Egrève; 4 Institut Universitaire de France, Paris; 5 38000, C3R – Réhabilitation psychosociale et remédiation cognitive, Centre Hospitalier Alpes Isère, Grenoble, France

## Abstract

**Introduction:**

Auditory Hallucinations (AH) can be distressing experiences lived by clinical samples but can also be observed in the general population. Predictive Coding Theories of AH argue that when strong priors are favoured over sensory input, AH would emerge. Powers and collaborators (2017) and Benrimoh et al. (2024) have employed the Conditioned Hallucinations task (CHT) to demonstrate that strong priors were linked to AH. In the CHT, conditioned hallucinations (CH) were created using tones, which neglects the fact that most patients describe AH as verbal and characterised by negative content. Consequently, little is known about the effect of the nature (i.e., verbal) and valence of the AH within the Predictive coding framework of hallucinations. More specifically, the role of emotional voice content in CH has not been explored.

**Objectives:**

Thus, our goal is to replicate and expand these results by manipulating the content valence of voices in the CHT. This will allow us to test a possible interaction effect of voice content and the proneness to AH on the rise of CH.

**Methods:**

We will recruit 400 French participants from the general population presenting different levels of proneness to AH. Participants will undergo an adapted version of the CHT with two different blocs where either negative or neutral voice content will be presented. Following Powers et al. (2017), for both the negative and neutral conditions, participants will undergo a QUEST maximum-likelihood-based procedure to derive individual thresholds. After, they will complete 12 blocs, during which the number of signals present and their volume will decrease. A no-signal condition will also be presented. The speech presented will be French translation of Baumeister et al. (2022) stimuli. They were created to simulate verbal AH. Since some voice content resemble inner dialogue, we will measure the inner dialogue forms of participants through the Forms of Self-Criticizing/Attacking & Self-Reassuring Scale. Hallucination proneness will be measured through the Launay and Slade Hallucination Scale Extended we modified. Our local ethical committee approved this study following the Helsinki and APA principles.

**Results:**

We are currently collecting data and are not able to communicate any results at this time. Data collection should be done by April 2025. Our data will be analyzed through the signal detection theory, a logistic regression on the probability of saying a signal is present and through Hierarchical Gaussian Filter Analysis.

**Conclusions:**

A better understanding of the mechanisms behind AH and the role of emotions will help us improve predictive coding theories of AH that can also used to improve interventions targeting them.

**Disclosure of Interest:**

None Declared

